# Diagnostic Utility and Pathogenic Role of Circulating MicroRNAs in Vasospastic Angina

**DOI:** 10.3390/jcm9051313

**Published:** 2020-05-02

**Authors:** Chan Soon Park, Inho Kim, Gyu Chul Oh, Jung-Kyu Han, Han-Mo Yang, Kyung Woo Park, Hyun-Jai Cho, Hyun-Jae Kang, Bon-Kwon Koo, Woo-Young Chung, Seil Oh, Hae-Young Lee

**Affiliations:** 1 Graduate School of Medical Science and Engineering, Korea Advanced Institute of Science and Technology, Daejeon 34141, Korea; shiny365@hanmail.net; 2 Department of Microbiology and Immunology, Seoul National University College of Medicine, Seoul 03080, Korea; inokio@gmail.com; 3 Department of Internal Medicine, Seoul National University Hospital, Seoul 03080, Korea; david.gyuchul@gmail.com (G.C.O.); hpcrates@gmail.com (J.-K.H.); hanname@hanmail.net (H.-M.Y.); kwparkmd@snu.ac.kr (K.W.P.); hyunjaicho@snu.ac.kr (H.-J.C.); nowkang@snu.ac.kr (H.-J.K.); bkkoo@snu.ac.kr (B.-K.K.); seil@snu.ac.kr (S.O.); 4 Department of Internal Medicine, Seoul National University College of Medicine; Seoul 03080, Korea; chungwy3023@daum.net; 5 Department of Internal Medicine, Seoul Metropolitan Government Seoul National University Boramae Medical Center, Seoul 07061, Korea

**Keywords:** vasospastic angina, microRNA, diagnosis, pathogenesis

## Abstract

We investigated the diagnostic value and pathophysiological role of circulating microRNA (miR) in vasospastic angina (VA). We enrolled patients who underwent coronary angiography for chest pain to explore the miR’s diagnostic utility. In addition, we investigated the role of miRs in regulating endothelial nitric oxide synthase (eNOS) expression in human coronary artery endothelial cells (hCAECs). Among the 121 patients, 46 were diagnosed with VA (VA group), 26 with insignificant coronary lesions (ICL group), and 49 with atherothrombotic angina (AA group). The VA group showed a significantly higher expression of miR-17-5p, miR-92a-3p, and miR-126-3p than the ICL group. In contrast, miR-221-3p and miR-222-3p were upregulated in the AA group compared to the VA group, and all levels of miR-17-5p, miR-92a-3p, miR-126-3p, miR-145-5p, miR-221-3p, and miR-222-3p differed between the AA group and the ICL group. In the hCAECs, transfection with mimics (pre-miR) of miR-17-5p, miR-92a-3p, and miR-126-3p was associated with eNOS suppression. Additionally, transfection with inhibitors (anti-miR) of miR-92a-3p significantly rescued the eNOS suppression induced by lipopolysaccharide. In conclusion, the circulating miRs not only proved to have diagnostic utility, but also contributed to pathogenesis by eNOS regulation.

## 1. Introduction

Vasospastic angina (VA), caused by dynamic occlusion of the major epicardial coronary artery, is characterized by resting chest pain accompanied by transient ST segment deviation on electrocardiography, but the coronary artery shows normal appearance on resting coronary artery angiography (CAG) [1,2]. Due to its characteristic pathophysiology, chest pain in VA does not occur in parallel with the increase of myocardial oxygen demand, and is often overlooked in practice.

VA prevalence varies worldwide, with approximately 40% in Japan, but less in Western countries, and presents a substantial burden among patients with angina [3,4]. In many cases, VA occurs in young or middle-aged patients who are considered to be at low risk of cardiovascular disease [5]; therefore, it is often difficult to distinguish VA from other causes through the medical history alone. However, clinical guidelines recommend considering VA not only in the aforementioned patients but also in those with persistent angina and in those with symptoms after percutaneous coronary intervention. The current standard diagnostic method is CAG with a pharmacologic provocation test using acetylcholine or ergonovine, which is known to have higher sensitivity than other tests [6,7]. However, its application has often been limited due to its invasiveness, especially for patients with a low VA probability. If patients with chest pain suspicious of coronary artery disease (CAD) and some possibility of VA were planned to undergo CAG, clinicians should contemplate the problem of whether to stop vasodilators before CAG. In the cases of those with chest pain but without a fixed atherothrombotic lesion in coronary angiography, the provocation test should be considered to rule out VA. However, patients with chest pain are often treated with vasodilators to relieve symptoms and signs of myocardial ischemia, and these patients have to reschedule the CAG with a provocation test after abstaining from vasodilators for 24–48 hours. Although noninvasive echocardiography with pharmacological provocation has been used in some institutes [8], the lack of coronary artery access can be life-threatening.

Human microRNAs (miRs) are small, single-stranded, endogenous noncoding RNAs that regulate gene expression at the post-transcriptional level by promoting the messenger RNA (mRNA) degradation or repressing certain coding mRNA translation [9]. The miRs are released from the cells to the tissues and blood, although their total fraction is relatively small [10,11]. The expression levels of certain miRs are altered in various pathological conditions such as malignancy, acute kidney injury, and CAD [12,13,14]. Several miRs have been reported to be associated with CAD; these include miR-17-5p, miR-92a-3p, miR-126-3p, miR-145-5p, miR-221-3p, and miR-222-3p, which have been reported to be associated with endothelial impairment or smooth muscle cell pathology [15,16]. Considering that endothelial dysfunction and smooth muscle cell hyper-reactivity have been suggested as pathologic coronary vasospasm mechanisms [17], we hypothesized that distinct circulating miR profiles could discriminate patients with VA from those without significant coronary artery lesions among patients with chest pain. Furthermore, we postulate that miRs might be associated with VA pathogenesis in the aspects of managing the endothelial cell function and controlling the smooth muscle reactivity.

Considering these observations together, we first examined the miR expression profiles related to the cardiovascular system, and investigated the miR diagnostic value in distinguishing patients with VA from those with insignificant coronary lesions (ICLs). After verification of the diagnostic potential of miR profiles, we aimed to investigate how circulating miRs could influence VA pathogenesis. We evaluated the impact of miRs in regulating the endothelial nitric oxide synthase (eNOS) expression using human coronary artery endothelial cells (hCAECs).

## 2. Materials and Methods

### 2.1. Patients

**Figure 1:**
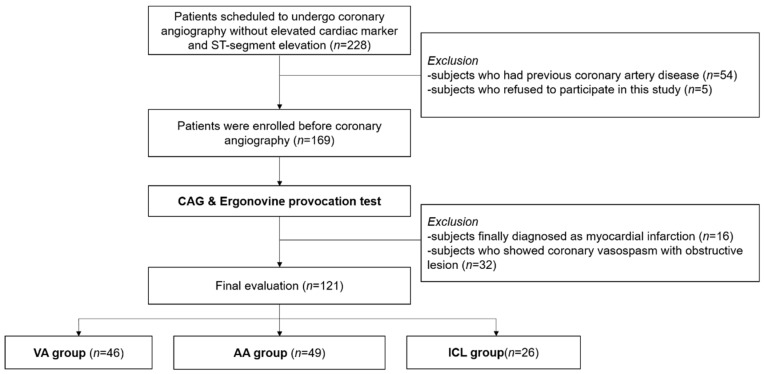
Patient enrolment flow. AA, atherothrombotic angina; CAG, coronary angiography; ICL, insignificant coronary lesion; VA, vasospastic angina.

We recruited patients who underwent CAG due to chest pain from 2013 to 2016 at Seoul National University Hospital. A flowchart of this study is presented in Figure 1. In both the outpatient clinic and emergency department, we screened patients scheduled to undergo CAG whom with anginal pain without elevated cardiac markers (high-sensitivity cardiac troponin level above the 99th percentile) and without ST segment elevation (*n* = 228). Medical history examination, physical examination, laboratory tests, ECG, and CAG were performed according to clinical guidelines [17,18,19,20]. Among screened individuals, we excluded patients who had been previously diagnosed with obstructive coronary artery disease (*n* = 54) or refused to participate in the study (*n* = 5). Patients who showed elevated cardiac markers at follow-up tests were also excluded (*n* = 32).

According to the results of CAG and provocation test, we categorized the patients into three groups—patients with significant coronary obstructive lesion and without coronary vasospasm (AA group), those without a coronary obstructive lesion and with coronary vasospasm (VA group), and those without any significant obstructive lesion or vasospasm (ICL group). The patients who showed no fixed lesions and negative results on provocation test on CAG were assigned to the ICL group. To avoid overlapped effects of obstructive lesion and coronary vasospasm, patients with both obstructive lesion and coronary vasospasm were excluded from this study (*n* = 32). These patients showed coronary vasospasm without ergonovine administration, and those with a fixed lesion with marginal significance were diagnosed with coronary vasospasm by provocation test results. Finally, we evaluated the expression patterns of miRs in 121 patients; 46 patients were diagnosed with VA, 26 patients with ICL, and 49 patients with AA. The study protocol was approved by the Institutional Review Board of the Seoul National University Hospital (E-1602-086-741; February 24th, 2016) and the study was conducted according to the principles of the Declaration of Helsinki. Written informed consent was obtained from all participants.

### 2.2. Data Collection from Study Participants

We collected demographic data, past medical history, and laboratory results. Blood sampling, excluding miRs, and other tests were conducted as routine practice by a laboratory center certified by the Korean Association of Quality Assurance for Clinical Laboratory.

The final diagnosis of typical chest pain was assessed by interventional cardiology specialists based on their symptoms and CAG data. Patients were categorized into three groups as follows—VA group, AA group, and ICL group.

### 2.3. Ergonovine Provocation Test

The diagnosis of VA was made based on the standard guidelines for diagnosis and treatment of VA [17]. In the current study, intracoronary ergonovine injection was adopted. At first, CAG was performed to find the best projection such that intervention cardiologists could discriminate coronary arteries clearly. Subsequently, 20 μg of ergonovine was injected into the left coronary artery at 5 min intervals. In cases of negative results, ergonovine was injected into the right coronary artery in a similar manner. After provocation, a sufficient dose of nitrate was administered to each coronary artery, and angiography was performed again for maximal dilation. Positive test results were defined as cases with transient, subtotal, or total occlusion (>90%) of a coronary artery with signs of myocardial ischemia (angina chest pain and ischemic ST changes). All calcium channel blockers or long-acting vasodilators were withdrawn more than two days before the provocation test.

### 2.4. Blood Sample Collection and miR Assay

Under sterile conditions, the blood was drawn immediately after the percutaneous guiding catheter reached the aorta during the CAG. We designed the study protocol to collect the blood before the ergonovine provocation and with minimal use of heparin to alleviate the possibility of confounding effects caused by coronary intervention, heparin application, or ergonovine provocation on miR analysis. In total, 5 mL of blood was collected into serum separation tubes and centrifuged at 2500 rpm at 4 °C for 10 min. The supernatant was transferred to RNase/DNAse-free tubes and stored at −196 °C until the miRs were analyzed. This storage was considered appropriate since several studies have shown that the miRs in frozen samples remain stable for years [21,22].

Total RNA was extracted and isolated from the serum or cell pellet using a commercially available kit (miRNeasy serum/plasma kit or miRNeasy mini kit, Qiagen, Valencia, CA, USA) according to the manufacturer’s instructions. Reverse transcription was then performed using the previously obtained RNA (miScript II RT kit, Qiagen, Valencia, CA, USA). We purchased predesigned commercially available primer sequences (miScript Primer assay, Qiagen, Valencia, CA, USA). RT-qPCR was performed in a reaction volume of 25 μL with the miScript SYBR green PCR kit according to the manufacturer’s instructions for each miR (Qiagen, Valencia, CA, USA). Quantification of RNA in each sample was performed using the ΔCt method. When analyzing circulating miRs in the serum, *Caenorhabditis elegans*-miR-39 (Ce-miR-39) was used as a spike-in internal standard. When analyzing miRs from cultured hCAECs, RNU6 was used as an internal control. Used primer sequences are presented in Table S1.

### 2.5. Cell Culture and Transfection

Human coronary artery endothelial cells (hCAECs) were purchased from Lonza (Basel, Switzerland) and were cultured in endothelial cell growth medium consisting of endothelial basal media and EGM-2MV Bullet Kit (Lonza, Basel, Switzerland) at 37 °C in an atmosphere of 95% air and 5% CO_2_. Culture media were replaced every 2 days, and the cells were used between passages 6 and 8. When the cells reached 70–80% confluency, a final concentration of 100 nM of synthetic miR mimics (Catalog No. 4464066; Invitrogen, Carlsbad, CA, USA) or anti-miRs (Catalog No. 4464084; Invitrogen, Carlsbad, CA, USA) was transfected into the cells with lipofectamine RNAiMAX reagent (Invitrogen, Carlsbad, CA, USA). At 6 hours post-transfection, the media were replaced, and the cellular lysates were collected 24 hours later for total protein or RNA isolation. A random sequence anti-miR (anti-miR-control, Ambion, Austin, TX, USA) or random sequence pre-miR (pre-miR-control, Ambion, Austin, TX, USA) was used as negative controls, respectively.

### 2.6. Western Blotting 

Cultured hCAECs were washed with ice-cold phosphate-buffered saline (PBS) and lysed in radioimmunoprecipitation assay buffer (RIPA) cell lysis buffer (Santa Cruz biotechnology, Santa Cruz, CA, USA) with protease inhibitor on ice for 15 min. The lysed cells were centrifuged at 13,000 rpm for 15 min at 4 °C to separate the proteins. The protein concentration was determined by the bicinchoninic acid assay (BCA) protein assay (Pierce Biotechnology, Rockford, IL, USA), and after boiling for 5 min at 95 °C with sample buffer, the protein samples (20–30 μg) were separated on 8% SDS-polyacrylamide gels and blotted onto polyvinylidene difluoride (PVDF) membranes (Immobilon-P; Millipore, Bedford, MA, USA). The membranes were then blocked with TBST containing 5% skim milk for 1 hour and incubated overnight at 4 °C in blocking buffer with polyclonal anti-eNOS (1:1000; Catalog No.4464058; Invitrogen, Carlsbad, CA, USA), anti-KLF2 (1:1000; Catalog No. PA5-40591; Invitrogen, Carlsbad, CA, USA), or anti-β-actin (1:2000; Santa Cruz Biotechnology, Santa Cruz, CA, USA) primary antibodies. The membranes were then washed in tris-buffered saline with tween-20 (TBST) and incubated with horseradish peroxidase-conjugated goat antirabbit (1:5000; Invitrogen, Carlsbad, CA, USA) or rabbit antigoat secondary antibodies (1:5000; Santa Cruz Biotechnology, Santa Cruz, CA, USA) for 1 hour at room temperature, before being washed again in TBST. The signal was detected using the chemiluminescence detection reagent ECL (Promega, Madison, WI, USA), and protein levels were acquired by densitometric scanning. The obtained values were expressed in arbitrary densitometric units and normalized to those of β-actin to correct for total protein loading.

### 2.7. Statistical Analysis

Data are presented as numbers and frequencies for categorical variables and as mean ± standard deviation or median with interquartile range for continuous variables. For comparison between groups, the χ^2^ test or Fisher’s exact test was used for categorical variables, and the unpaired Student’s t-test was applied for continuous variables. One-way analysis of variance and Scheffé’s post-hoc test were used to analyze differences for continuous variables among more than two groups. We calculated the area under the curve (AUC) by receiver operating characteristics (ROC) analysis to evaluate the predictive value of miRs for VA, AA, and ICL. Correlations between specific miRs and other cardiovascular risk factors were analyzed by multivariate analysis and reported as Spearman’s correlation coefficients. Univariate and multivariate logistic regression models were employed to identify miRs associated with CAG results. Two-sided *p*-values < 0.05 were considered significant. Statistical tests were performed using the STATA software (version 12, Stata Corp., College Station, Texas, USA).

## 3. Results

### 3.1. Baseline Characteristics of Subjects

The baseline characteristics of the included patients are summarized in Table 1. Briefly, patients with VA were young and the AA group showed the highest male preponderance. There was no significant difference in the prevalence of cardiovascular risk factors, such as hypertension, hyperlipidemia, chronic kidney disease, and smoking history, while the AA group showed high prevalence of diabetes mellitus. The use of aspirin was frequently observed in the AA group, and the use of lipid-lowering agents was equivalent across groups.

### 3.2. MiR Expression Profiles

**Figure 2:**
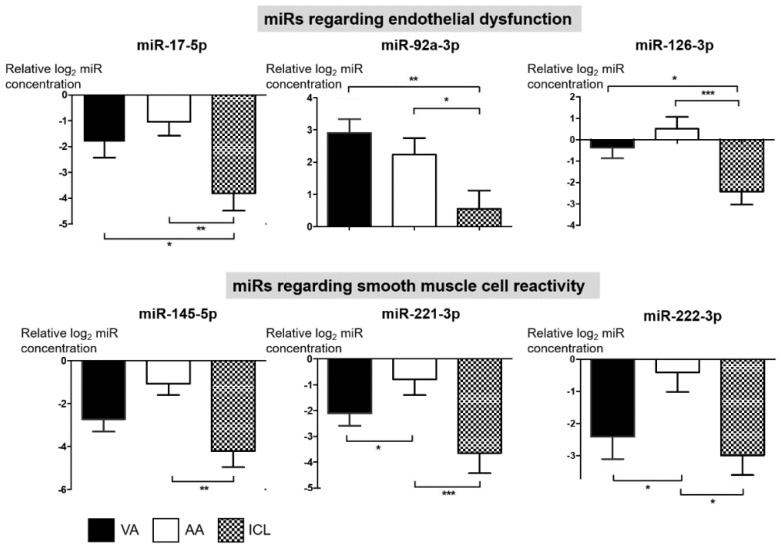
Circulating miR profiles according to coronary artery disease category. The miR expression relative to mean of the control miR, c-elegans-39 (miR-39) in the VA group (*n* = 46), AA group (*n* = 49), and ICL group (*n* = 26) is demonstrated. *Y*-axis scale is log-transformed. AA, atherothrombotic angina; Ct, cycle threshold; ICL, insignificant coronary lesion; miR, microRNA; VA, vasospastic angina. One-way analysis of variance was applied and data are presented as mean ± standard deviation. * indicates *p* < 0.05, ** indicates *p* < 0.01, *** indicates *p* < 0.001.

The miR expression patterns of patients with VA and those with ICL were analyzed. In patients with VA, the concentration of miRs pertaining to endothelial dysfunction was significantly higher than that in patients with ICL (Figure 2, miR-17-5p, miR-92a-3p, and miR-126-3p). However, miRs pertaining to smooth muscle cell reactivity showed serum concentrations comparable to those of the ICL group (Figure 2, miR-145-5p, miR-221-3p, and miR-222-3p). The threshold cycle (Ct) values of each miR are shown in Table S2. Of note, the levels of miRs pertaining to endothelial dysfunction were similar between the VA group and AA group (Figure 2, miR-17-5p, miR-92a-3p, and miR-126-3p), while significant differences between the two groups were observed in miRs pertaining to smooth muscle cell reactivity (Figure 2, miR-221-3p and miR-222-3p). Indeed, the AA group showed significantly higher concentrations of all six miRs than the ICL group (Figure 2); this is consistent with a previous study, which reported miR alterations in patients with coronary endothelial dysfunction [14]. The discriminative power of miRs in identifying the VA group from the summation of other groups is presented in Figure S1.

Next, we investigated the association between the miR serum concentrations and other covariates using multivariate analysis (Table 2). Age and sex, as well as levels of hemoglobin, leukocytes, creatinine, total cholesterol, high-density lipoproteins, and high-sensitivity C-reactive proteins were exclusively adjusted in each analysis. Among these, the miR concentrations generally did not demonstrate a significant relationship, with the exception of miR-221-3p and leukocyte count (*r* = 0.213, *p* = 0.015), and miR-17-5p and low-density lipoprotein (*r* = 0.220, *p* = 0.015).

### 3.3. MiR Score to Discriminate Patients with VA from Those with ICL

**Figure 3:**
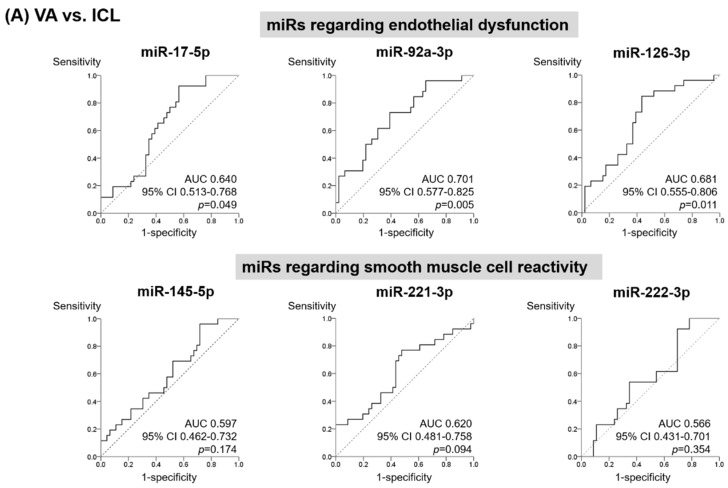

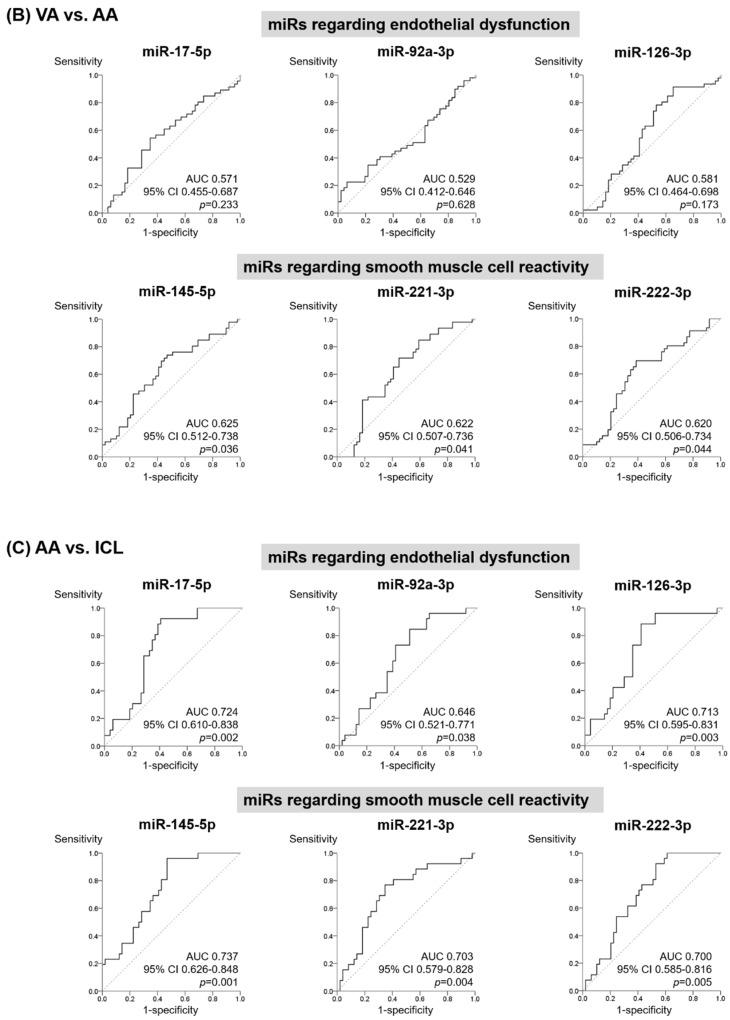
Distinction of patients according to miR profile. In the receiver operating characteristics curve analysis, the area under the curve and 95% confidence intervals were calculated between (**A**) VA group (*n* = 46) group and ICL groups (*n* = 26), (**B**) VA group and AA group (*n* = 49), and (**C**) AA group and ICL group. AA, atherothrombotic angina; AUC, area under the curve; CI, confidence interval; ICL, Insignificant coronary lesion; VA, vasospastic angina; miR, microRNA.

We evaluated the circulating miR diagnostic utility using receiver operating characteristics analysis. As demonstrated, miR-17-5p (area under the curve (AUC), 0.640; 95% confidence interval (CI), 0.513–0.768; *p* = 0.049), miR-92a-3p (AUC, 0.701; 95% CI, 0.577–0.825; *p* = 0.005), and miR-126-3p (AUC, 0.681; 95% CI, 0.555–0.806; *p* = 0.011) had the power to discriminate patients with VA and ICL (Figure 3A). On the contrary, miR-145-5p (AUC, 0.597; 95% CI, 0.462–0.732; *p* = 0.174), miR-221-3p (AUC, 0.620; 95% CI, 0.481–0.758; *p* = 0.094), and miR-222-3p (AUC, 0.566; 95% CI, 0.431–0.701; *p* = 0.354) did not show significant diagnostic value when analyzed separately. In addition, we explored the diagnostic utility of circulating miRs in discriminating between the AA and VA groups (Figure 3B) and the AA and ICL groups (Figure 3C), and miR profiles also showed discriminative power to distinguish these groups.

### 3.4. Functional Role of miRs in Regulating Endothelial Function

**Figure 4:**
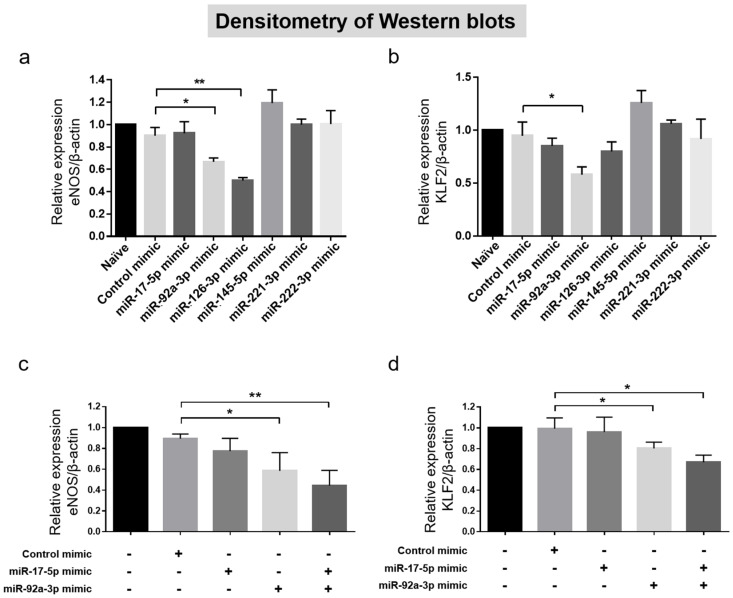
The role of miRs in endothelial eNOS protein expression. Densitometry of eNOS after each pre-miR transfection (*n* = 3) (**A**), densitometry of KLF2 after each pre-miR transfection (*n* = 3) (**B**), and densitometry of eNOS and KLF2 after pre-miR-17-5p and pre-miR-92a-3p co-transfection (*n* = 3) (**C**,**D**). One-way analysis of variance was applied and data are presented as mean ± standard deviation. * indicates *p* < 0.05, ** indicates *p* < 0.01, *** indicates *p* < 0.001. eNOS: Endothelial nitric oxide synthase; miR, microRNA.

After analyzing the miR clinical implications in patients with VA, we performed an in vitro assay using hCAECs to explore the mechanisms of the relationship between miRs and VA. We transfected hCAECs with each pre-miR separately to increase the levels of miRs and evaluate the changes in eNOS expression levels in each transfection. Compared to the control (Catalog No.4464058; Invitrogen, Carlsbad, CA, USA), the eNOS expression level decreased significantly when pre-miR-92a-3p or pre-miR-126-3p was transfected into the hCAECs (Figure 4A and Figure S2A). Regarding the KLF2 levels, which acts upstream of eNOS [23], pre-miR-92a-3p transfection showed a significant decrement, while other miRs did not (Figure 4B and Figure S2B). In contrast, the eNOS levels did not decrease when pre-miR-17-5p, pre-miR-145-5p, pre-miR-221-3p, or pre-miR-222-3p were transfected into hCAECs. Additionally, we revealed that combined transfection of pre-miR-17-5p and pre-miR-92a-3p into hCAECs led to additive decreases of eNOS and KLF2 expression levels, compared to transfections with either pre-miR-17-5p or pre-miR-92a-3p alone (Figure 4C,D and Figure S2C).

### 3.5. Rescue of Endothelial Dysfunction when Treated with AntagomiRs

**Figure 5:**
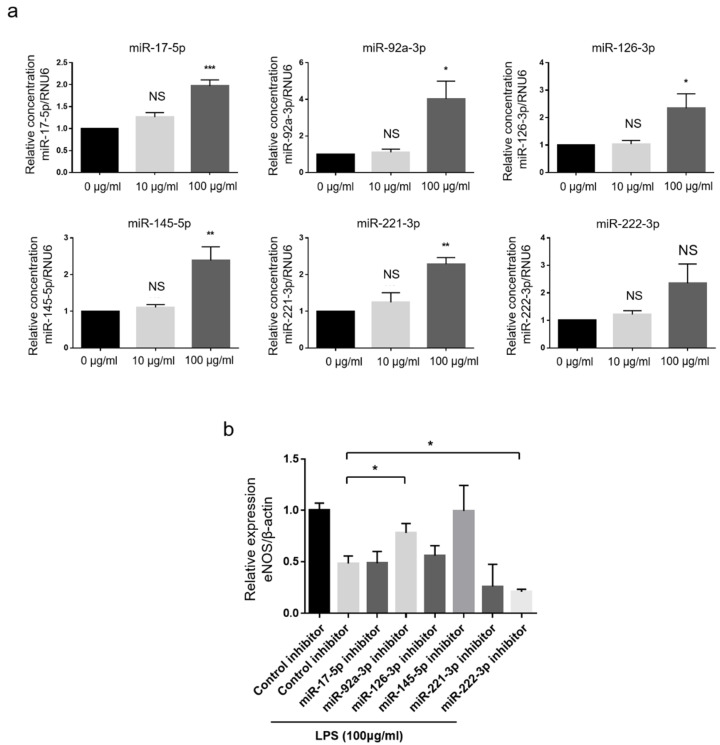
Role of miR modulation in eNOS protein expression in hCAECs treated with the vasculotoxic stimuli, LPS. (**A**) Relative concentration of miR-17-5p, miR-92a-3p, miR-126-3p, miR-145-5p, miR-221-3p, and miR-222-3p in hCAECs according to LPS concentration (*n* = 3). (**B**) Densitometry of eNOS after transfection of each anti-miR-17-5p, anti-miR-92a-3p, anti-miR-126-3p, anti-miR-145-5p, anti-miR-221-3p, and anti-miR-22-3p (*n* = 4). One-way analysis of variance was applied and data are presented as mean ± standard deviation. * Indicates *p* < 0.05, ** indicates *p* < 0.01, and *** indicates *p* < 0.001 compared to the control group with LPS treatment. eNOS, endothelial nitric oxide synthase; hCAECs, human coronary artery endothelial cell; LPS, lipopolysaccharide; miR, microRNA; ns, not significant

We evaluated whether anti-miR-17-5p or anti-miR-92a-3p could improve endothelial dysfunction by restoring eNOS downregulation. The hCAECs were treated with lipopolysaccharide (LPS), a well-known toxic stimulus to the endothelial cells [24], in order to consequently induce eNOS suppression and endothelial dysfunction. The eNOS expression level in hCAECs was significantly suppressed by LPS treatment at doses greater than 10 μg/mL (Figure S3). The expression of miR-17-5p, miR-92a-3p, miR-126-3p, miR-145-5p, and miR-221-3p was significantly increased after LPS treatment at 100 μg/mL (Figure 5A). Interestingly, eNOS expression suppression by LPS was significantly reversed when hCAECs were transfected with anti-miR-92a-3p (Figure 5B and Figure S4).

## 4. Discussion

The major findings of this study are as follows—(1) the circulating miR levels were different between the patients with VA and those with ICL; the VA group showed significantly higher expression levels of miR-17-5p, miR-92a-3p, and miR-126-3p than the ICL group and equivalent expression levels of miR-145-5p, miR-221-3p, and miR-222-3p; (2) the expression levels of the miRs were generally independent of other cardiovascular risk factors; (3) the miR profiles showed discriminatory power in identifying the VA group from other groups; (4) miR-92a-3p and miR-126-3p separately influenced eNOS expression in the hCAECs, and miR-17-5p showed additive effects when co-transfected with miR-92a-3p; (5) antago-miR of miR-92a-3p reversed the decrease in eNOS expression when it was suppressed in pathological conditions.

### 4.1. Association Between miRs, Endothelial Dysfunction, and VA

The VA etiology is multifactorial; however, endothelial dysfunction and smooth muscle cell hyper-reactivity are considered as the two main mechanisms [16,25]. With the release of vasodilators such as nitric oxide (NO), the endothelial cells play a pivotal role in coronary vascular tone regulation [26]. In normal conditions, the acetylcholine and ergonovine, which are used in the provocation test for VA, induce vasodilation by releasing vasodilators from the ‘healthy’ endothelial cells. However, in pathological conditions, these drugs result in paradoxical vasoconstriction [27,28]. Several studies have reported that dysfunctional NO synthase is related to coronary vasospasm [4,29,30]. Moreover, endothelial dysfunction has been observed even in nonspastic arteries of patients with VA, suggesting that endothelial dysfunction might be the main VA mechanism [31,32].

MiRs are significant regulators of gene expression by promoting degradation and inhibiting the target mRNAs translation. Various miRs have been studied in cardiovascular diseases [9]; among them, miR-17-5p, miR-92a-3p, and miR-126-3p have been reported to be associated with endothelial function. Their expression has been studied in atherosclerosis, angiogenesis, endothelial cell differentiation, and leukocyte trafficking during inflammation [33,34,35,36]. Therefore, we postulated that these miRs could have an impact on endothelial dysfunction in patients with VA. Indeed, the expression levels of these miRs were increased in the VA group compared to those in the ICL group, suggesting their involvement in endothelial dysfunction mediation. Intriguingly, the concentrations of miR-17-5p, miR-92a-3p, and miR-126-3p were similar to those of the AA group, in which the concentration of this miR cluster was reported to be increased [15].

### 4.2. Implication of miRs in the VA Pathophysiology

Endogenous NO exerts various actions in the vascular system, and previous studies have demonstrated that NO produced by eNOS has various vasculoprotective effects including vasodilation, platelet aggregation inhibition, low-density lipoprotein oxidation, and vascular smooth muscle cell proliferation [37,38]. In endothelial dysfunction cases, impaired NO production leads to vasoconstriction [16]. Although a higher prevalence of genetic mutations in the eNOS gene has been found among patients with VA [39], there is a lack of studies on its post-transcriptional regulatory mechanism. In this study, our data indicated that miR-92a-3p and miR-126-3p inhibited eNOS expression, suggesting that certain miRs might have a crucial role in VA pathogenesis.

Smooth muscle hyper-reactivity is another pathologic key feature of VA. The contraction and relaxation of vascular smooth cells are regulated by phosphorylation and dephosphorylation of the myosin light chains [40]. In porcine experiments, increased Rho-kinase activity enhanced coronary artery vasospasm [33,41,42]. Moreover, it has been shown that dysfunction of K_ATP_ channels is highly associated with smooth muscle cell hypercontraction in the absence of atherosclerosis [43,44]. In mouse models, the calcium channel influences vascular relaxation [45]. Taken together, we hypothesized that the abnormal smooth muscle cells might develop coronary artery spasm; however, this relationship in humans remains unclear and requires further investigation [25]. In previous reports, miR-145-5p, miR-221-3p, and miR-222-3p have been reported to be associated with the vascular smooth muscle cells [45,46]. Their expression levels in the AA group were significantly elevated compared to those in the ICL group, as previously reported [14]. However, the expression levels of these miRs in the VA group were not significantly different from those in the ICL group. Moreover, the VA group showed significantly lower concentrations of miR-145-5p and miR-222-3p than the AA group, suggesting that the miR profiles in patients with VA might support endothelial cell dysfunction rather than smooth muscle cell hyper-reactivity.

### 4.3. Mechanism of the Relationship Between miRs, eNOS, and VA

In addition to the clinical implications of miR in patients with VA, we found that several specific miRs could induce inhibition of eNOS expression, which exerts vasculoprotective effects by producing endogenous NO. We also investigated whether antago-miRs of these miRs could rescue the downregulation of eNOS expression. We did not explore the sophisticated biological pathways by which miRs regulate eNOS expression, but we can cautiously suggest one possible explanation for our results. Using TargetScan [47], a computational algorithm to predict miR targets, we found that miR-17-5p and miR-92a-3p did not directly target eNOS (Figure S5), but could act on the *KLF2* gene, which is reported to act as an upstream signal of eNOS [23]. Indeed, pre-miR-17-5p did not affect the expression levels of eNOS and expression, but co-transfection with pre-miR-17-5p and pre-miR-92a-3p synergistically downregulated the expression levels of eNOS and KLF2 in the hCAECs. Further studies are needed to reveal the elaborate molecular pathways involved in this process.

### 4.4. Clinical Implications of this Study

Our study suggests that circulating miR profiles could be novel biomarkers in diagnosing VA. In addition, we also performed an in vitro study using hCAECs to provide the hypothetical background for understanding VA pathogenesis. Considering these together, this study provides valuable information regarding the association between VA and miR profiles.

A recent study reported a scoring system that showed remarkable accuracy in diagnosing coronary artery vasospasm [48]. It is difficult to directly compare the result of our manuscript to those of the aforementioned studies due to differences in the baseline characteristics of patients and the definition of inclusion/exclusion criteria. However, it would be of interest to evaluate whether circulating miRs could serve as a novel biomarker beyond this system for diagnosing VA in further studies.

### 4.5. Limitations

Our study has several limitations. First, we used blood samples from the aorta, a representative sample of arterial blood, rather than from the coronary sinus [14,49,50]. However, our study was designed to show whether circulating miR profiles can distinguish patients with VA without additional invasive procedures, such as coronary sinus sampling. Secondly, we excluded patients who had coronary artery stenosis combined with coronary vasospasm. This group of patients was recently reported to have worse prognosis than those with coronary vasospasm only [5]. Though VA could be combined with fixed lesions, we excluded this group because of confounding effects of coronary artery pathologies on miR profiles. In addition, as we performed a provocation test using 20 μg of ergonovine, there is a possibility of underestimation of VA. Furthermore, this study was performed based on a single tertiary medical institute. The present study provides the evidence of association between the VA and circulating miR profiles, but the sample size is small and we could not verify how it could be functionally applied in practice. Therefore, a large multicenter study will be required to extensively evaluate the use of miRs as practical biomarkers. Finally, we thoroughly analyzed the expression patterns of miR-17-5p, miR-92a-3p, miR-126-3p, miR-145-5p, miR-221-3p, and miR-222-3p in this study. According to previous studies regarding coronary artery disease, we classified these miRs as those associated with endothelial dysfunction and those related to smooth muscle hypersensitivity. However, these miRs also showed discriminative powers and had different pathophysiologic roles in other cardiovascular diseases [51,52,53]. Thereby, interpreting the roles of miRs in cardiovascular diseases might require careful consideration. In addition, several miRs such as miR-16 were also suggested to have important role in cardiovascular diseases [54]. Although this study did not evaluate the role of miR-16, the next study should broaden the scope with more miRs, including miR-16 in VA.

## 5. Conclusions

Our study showed that the circulating miR profiles were different between the VA group and AA group and between the VA group and ICL group. In addition, the miR profiles showed discriminatory power in identifying the VA patients from others. We conclude that analyzing miR profiles could have diagnostic implications, although future studies are needed to establish cut-off values. Moreover, we demonstrated that miR-17-5p, miR-92a-3p, and miR-126-3p were associated with eNOS expression in hCAECs. We concluded that miRs could have a substantial clinical implication in patients with VA, not only as diagnostic markers, but also a pathophysiological role via eNOS regulation.

## Figures and Tables

**Table 1:** Baseline characteristics stratified by final diagnosis after coronary angiography.

Total (*n* = 121)	VA (*n* = 46)	AA (*n* = 49)	ICL (*n* = 26)	*p* Value
Demographic Data				
Age (Year) ^b^	59.2 ± 9.4	66.8 ± 9.0	65.1 ± 10.1	<0.001
Gender (Men, %) ^c^	31 (67.4)	39 (79.6)	13 (50.0)	0.031
Risk Factors (%)				
Hypertension	24 (52.2)	34 (69.4)	19 (73.1)	0.116
Diabetes Mellitus ^b^	6 (13.0)	22 (44.9)	5 (19.2)	0.001
Hyperlipidemia	23 (50.0)	35 (71.4)	16 (61.5)	0.101
Chronic Kidney Disease	1 (2.2)	1 (2.0)	2 (7.7)	0.369
Smoking				0.432
Never Smoker	33 (71.7)	15 (57.7)	15 (57.7)	
Current Smoker	7 (15.2)	5 (19.2)	5 (19.2)	
Ex-Smoker	6 (13.0)	6 (23.1)	6 (23.1)	
Medication at Admission				
Aspirin ^a,c^	10 (21.7)	23 (46.9)	6 (23.1)	0.017
Lipid-Lowering Agent	24 (52.2)	35 (71.4)	17 (65.4)	0.145
Laboratory Findings				
Hemoglobin (g/dL)	13.8 ± 2.0	13.9 ± 1.4	13.0 ± 1.6	0.057
Leukocyte (/μL)	6676.1 ± 1878.4	6928.0 ± 1535.1	6345.4 ± 1863.7	0.386
Platelet (10^3^/μL)	232.5 ± 62.3	231.3 ± 64.5	224.9 ± 51.3	0.869
Creatinine (mg/dL)	0.9 ± 0.2	0.9 ± 0.2	0.8 ± 0.2	0.107
Total Cholesterol (mg/dL)	143.5 ± 26.2	149.8 ± 38.4	144.2 ± 24.6	0.579
HDL Cholesterol (mg/dL) ^b^	49.8 ± 14.7	41.5 ± 14.3	48.6 ± 11.6	0.010
Triglyceride (mg/dL)	112.0 ± 57.8	128.7 ± 100.9	112.7 ± 57.7	0.526
LDL Cholesterol (mg/dL)	76.8 ± 23.4	80.8 ± 35.7	77.3 ± 23.5	0.797
hs-CRP (mg/dL)	0.06 (0.03–0.11)	0.11 (0.06–0.24)	0.10 (0.03–0.20)	0.507

HDL, high density lipoprotein; hs-CRP, high sensitivity C-reactive protein; LDL, low density lipoprotein. Post-hoc analysis was performed when the p value was <0.05. ^a^ Indicates *p* < 0.05 between the VA group and ICL group, ^b^ indicates *p* < 0.05 between VA group and AA group, and ^c^ indicates *p* < 0.05 between the AA group and ICL group.

**Table 2:** Association between cardiovascular risk factors and miR expression.

Risk Factors	miR-17-5p	miR-92a-3p	miR-126-3p	miR-145-5p	miR-221-3p	miR-222-3p
Age (Years)	−0.011	−0.010	0.021	0.063	0.146	0.041
Hemoglobin, (mg/dL)	0.127	0.089	0.087	0.119	−0.079	0.024
Leukocyte, (/uL)	−0.060	−0.055	0.019	−0.105	0.213 *	0.006
Creatinine (mg/dL)	0.034	0.031	0.090	−0.110	0.052	−0.051
Total Cholesterol (mg/dL)	0.058	0.065	0.155	−0.037	0.070	0.023
HDL Cholesterol (mg/dL)	0.139	0.032	−0.003	0.036	−0.179	−0.053
Triglyceride (mg/dL)	0.001	−0.118	−0.090	−0.044	0.007	0.002
LDL Cholesterol (mg/dL)	−0.220 *	−0.177	0.009	0.069	0.175	0.045
hs-CRP (mg/dL)	0.005	−0.034	−0.055	<0.001	−0.097	−0.055

Multivariable analysis of miR levels and cardiovascular risk factors. Values were adjusted for the cardiovascular risk factors, which were age, hemoglobin, leukocytes, creatinine, total cholesterol, HDL cholesterol, triglycerides, and LDL cholesterol. Spearman’s rank correlation coefficients are given with asterisks indicating significant positive or negative correlations set by a *p* < 0.05 (*). The expression of c-elegans-miR-39 was used as the internal standard. HDL, high density lipoprotein; LDL, low density lipoprotein; hs-CRP, high sensitivity C-reactive protein; miR, microRNA.

## Supplementary Materials

The following are available online at https://www.mdpi.com/2077-0383/9/5/1313/s1, Figure S1: Distinction of patients according to miR profile between VA group and other groups, Figure S2: The role of miRs in endothelial eNOS and KLF2 protein expression, Figure S3: eNOS protein expression in hCAECs after LPS treatment, Figure S4: The role of miR modulation in eNOS protein expression in hCAECs treated with LPS, Figure S5: Target scan of KLF2 gene, Table S1: Primer sequence, Table S2: Plasma miRNA levels at baseline detected by RT-qPCR.
