# Supplementary material for: Diagnostic Utility and Pathogenic Role of Circulating MicroRNAs in Vasospastic Angina
Source: J Clin Med. 2020 May 2;9(5):1313. doi: 10.3390/jcm9051313 (PMC7290712; doi:10.3390/jcm9051313)
Supplement: Supplementary file 1 [file jcm-09-01313-s001.pdf]

Supplementary material

Figure S1. Distinction of patients according to miR profile between VA group and other groups

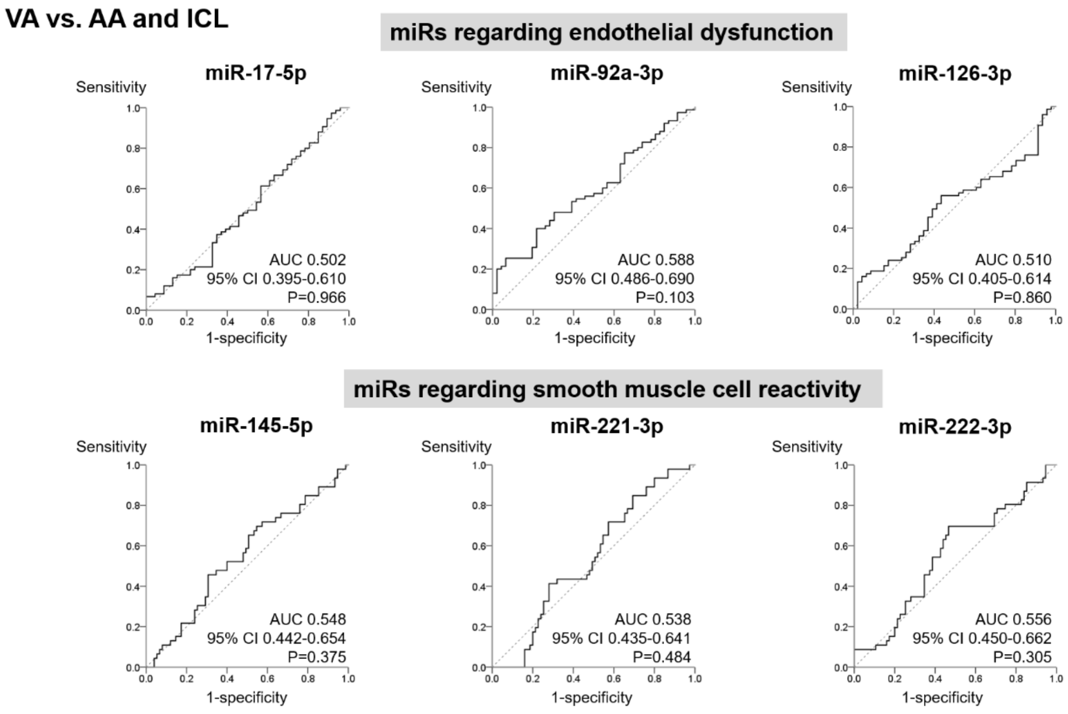

In receiver operating characteristics curve analysis, the area under the curve and 95% confidence interval were calculated.

VA: Vasospastic angina, miR: MicroRNA

Figure S2. The role of miRs in endothelial eNOS and KLF2 protein expression

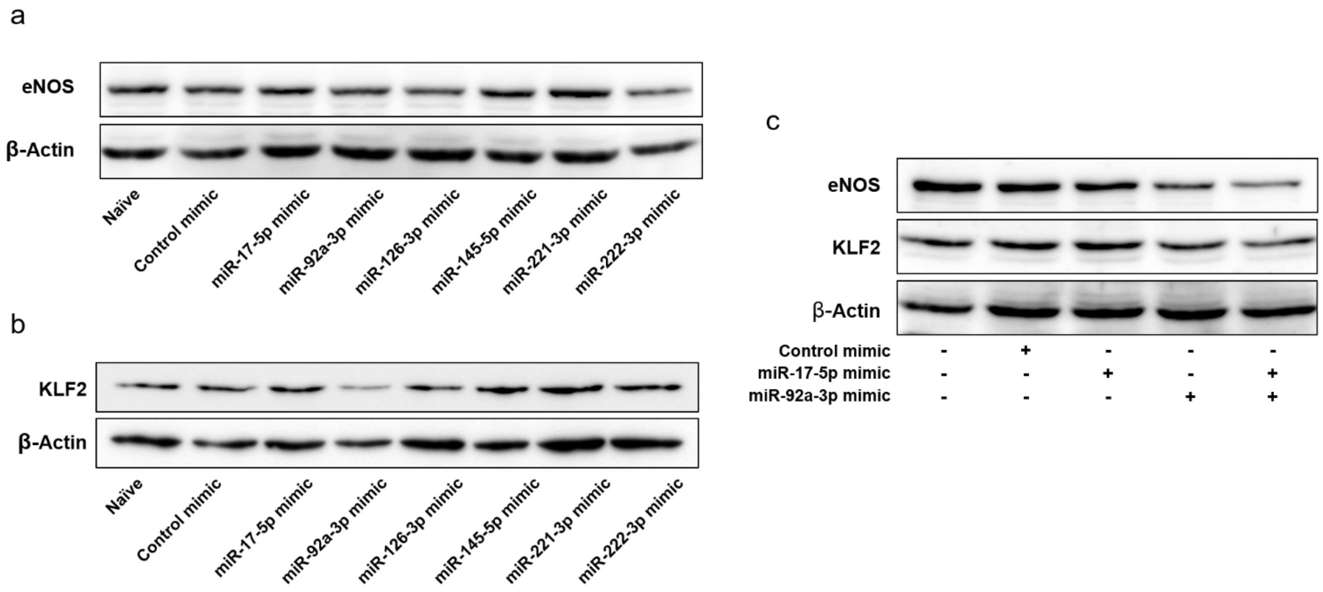

Western blot of eNOS after pre-miR transfection (A), western blot of KLF2 after pre-miR transfection (B), and western blot of eNOS and KLF2 after pre-miR-17-5p and pre-miR-92a-3p cotransfection (C).

eNOS, KLF2, and β-actin were analyzed in each single gel without cropping.

eNOS, endothelial nitric oxide synthase; miR, microRNA

Figure S3. eNOS protein expression in hCAECs after LPS treatment

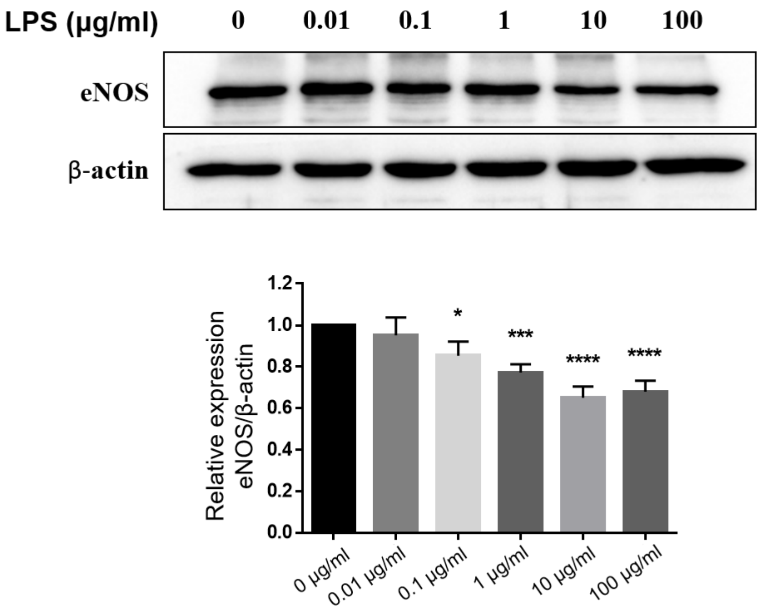

Western blot and densitometry of eNOS after LPS treatment. eNOS and β-actin were analyzed in each single gel without cropping.

One-way analysis of variance was applied and data are presented as mean ± standard deviation.

* indicates $p < 0.05$, ** indicates $p < 0.01$, *** indicates $p < 0.001$, and *** indicates $p < 0.001$.

eNOS, endothelial nitric oxide synthase; hCAEC, human coronary artery endothelial cell; LPS, lipopolysaccharide

Figure S4. The role of miR modulation in eNOS protein expression in hCAECs treated with LPS

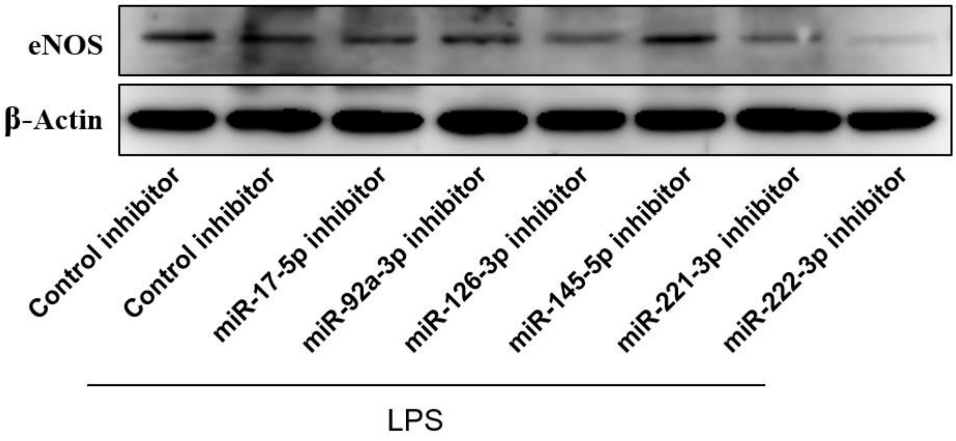

Western blot of eNOS after transfection of each antago-miRs. eNOS, and β-actin were analyzed in each single gel without cropping.

eNOS, endothelial nitric oxide synthase; hCAECs, human coronary artery endothelial cell; LPS, lipopolysaccharide; miR, microRNA.

Figure S5. Target scan of KLF2 gene

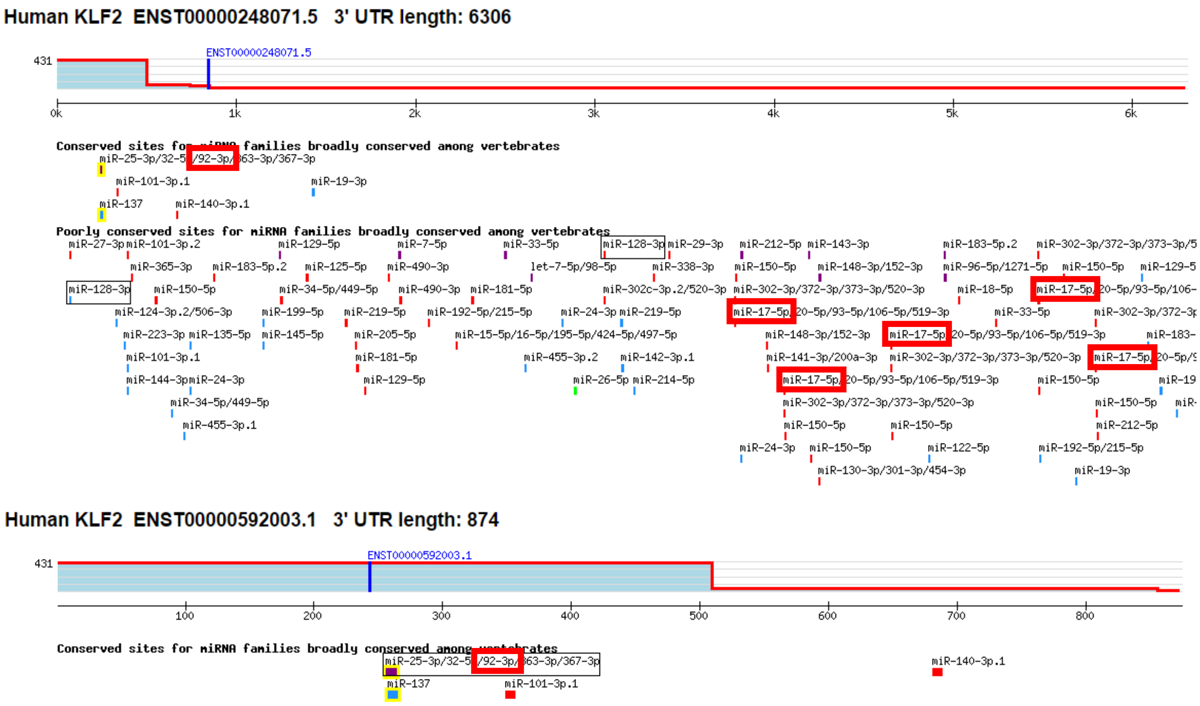

Table S1. Primer sequence

Primer	Sequence
eNOS forward	5'-GAAGGCTTTTGATCCCCGGGTCCTG-3'
eNOS reverse	5'-CAGTTCCTCCAGCCGTGTGTCCAC-3'
RNU6	5'-CGCAAGGATGACACGCAAATTC-3'
Hsa-miR-17-3p	5'-CAAAGUGCUUACAGUGCAGGUAG-3'
Hsa-miR-92a-3p	5'-UAUUGCACUUGUCCCGGCCUGU-3'
Hsa-miR-126-3p	5'-UCGUACCGUGAGUAAUAAUGCG-3'
Hsa-miR-145-5p	5'-GUCCAGUUUUCCCAGGAAUCCCU-3'
Hsa-miR-221-3p	5'-AGCUACAUUGUCUGCUGGGUUUC-3'
Hsa-miR-222-3p	5'-CUCAGUAGCCAGUGUAGAUCCU-3'
Ce-miR-39-3p	5'-UCACCGGGUGUAAAUCAGCUUG-3'

miR, microRNA

Table S2. Plasma miRNA levels at baseline detected by RT-qPCR.

	**Ct value by RT-qPCR**						
Risk factors	miR-17	miR-92	miR-126	miR-145	miR-221	miR-222	c-elegans-39
Total	33.4±3.3	29.4±4.3	31.9±3.2	33.9±3.3	33.5±3.6	33.0±3.9	31.5±4.7
VA	32.9±3.6	28.2±4.4	31.5±3.4	33.8±4.1	33.3±3.9	33.0±4.4	31.1±4.9
AA	34.4±2.7	31.1±3.6	32.8±2.4	34.4±2.5	34.2±2.8	33.7±3.2	33.3±3.6
ICL	32.5±3.7	28.1±4.4	31.1±3.7	32.9±3.0	32.3±4.0	31.7±3.9	28.7±4.7

AA, atherothrombotic angina; Ct, threshold cycle; miR, microRNA; ICL, insignificant coronary lesion; RT-qPCR, reverse transcription-quantitative real-time polymerase chain reaction; VA, vasospastic angina
